# Exploring half-metallic ferromagnetism and thermoelectric properties of Tl_2_WX_6_ (X = Cl and Br) double perovskites

**DOI:** 10.1039/d4ra02465f

**Published:** 2024-06-10

**Authors:** Ghulam M. Mustafa, Zaka Ullah, M. Adil Ameer, N. A. Noor, Sohail Mumtaz, Mohammad K. Al-Sadoon

**Affiliations:** a Department of Physics, Division of Science and Technology, University of Education Lahore Punjab 54770 Pakistan adilameer7673@gmail.com; b Department of Physics, Riphah International University Campus Lahore 53700 Pakistan; c Electrical and Biological Physics, Kwangwoon University Seoul 01897 South Korea; d Department of Zoology, College of Science, King Saud University P.O. Box 2455 Riyadh 11451 Saudi Arabia

## Abstract

Half-metallic semiconductors typically exhibit 100% spin polarization at the Fermi level which makes them desired materials for spintronic applications. In this study, we reported a half-metallic ferromagnetic nature in vacancy-ordered double perovskites Tl_2_WX_6_ (X = Cl and Br). The magnetic, electronic, and thermoelectric properties of the material are studied by the use of density functional theory (DFT). For the calculations of exchange–correlation potential, PBE-sol is employed while more accurate electronic band structure and density of states (DOS) are calculated by the mBJ potential. Both materials exhibited structural stability in the cubic structure with *Fm*3̄*m* space-group. The mechanical stability is confirmed by their computed elastic constants while their thermodynamic stability is attested by negative formation energy. The spin-based volume optimization suggested the ferromagnetic nature of the materials which is further confirmed by the negative value of the exchange energy *Δ*_x_(pd). Moreover, computed magnetic moment value for Tl_2_WCl_6_ and Tl_2_WBr_6_ is 2 μB and the majority of this comes from W. The spin-polarized band structure and DOS confirmed that both materials are half-metallic and at the Fermi level they exhibit 100% spin polarization. Furthermore, in the spin-down state, materials behave as semiconductors with wide bandgaps. Lastly, the thermoelectric properties are evaluated by the BoltzTrap code. The thermoelectric parameters which include the Seebeck coefficient, electrical conductivity, thermal conductivity, power factor, and figure of merit (*ZT*) are investigated in the range of temperatures from 200 to 800 K. The half-metallic ferromagnetic and thermoelectric characteristics make these materials desired for spintronics and thermoelectric applications.

## Abstract 2

Half-metallic semiconductors typically exhibit 100% spin polarization at the Fermi level which makes them desired materials for spintronic applications.

## Graphical abstract

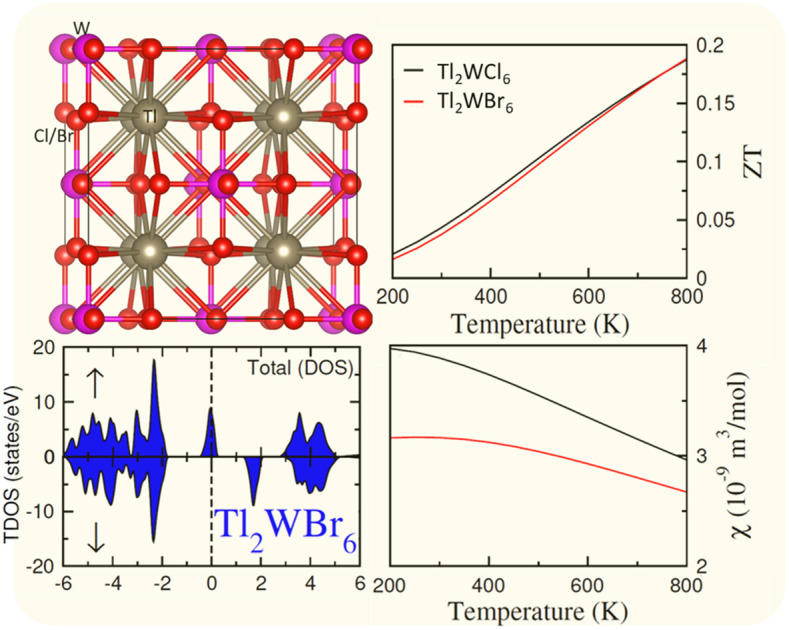

## 1. Introduction

Spintronics is a growing field of magnetic electronics, that has the potential to revolutionize the world by replacing conventional electronics. In conventional electronics, only the charge of electrons is accounted for while spintronics also utilizes the spin degree of freedom.^1^ In 1988, the discovery of giant magnetoresistance (GMR) in multilayer structures consisting of a nonmagnetic conducting layer sandwiched between ferromagnetic magnetic layers provided the foundation for this novel idea of spintronics.^2^ Furthermore, a high tunneling magnetoresistance (TMR) at room temperature was reported in 1995 which makes this phenomenon attractive for the development of magnetic sensors and memory storage devices.^3^ In addition, other advanced spin-based devices include spin transistors, spin capacitors, spin valves, and spin lasers.^4^ The advantages of spintronics over conventional electronics are their high-density memory and logic, high speed, and low power consumption^5^ with unlimited endurance.^6^

Spintronics works by creating spin polarization, and materials that provide high spin polarization are considered desirable.^7^ In this regard, several materials which include half metals (HM), ferromagnetic semiconductors (FMS), and dilute magnetic semiconductors (DMS) are considered suitable as HM and FMS provide high spin polarization and DMS provide ease for spin injection.^8–10^ In recent decades, researchers have discovered both half metallic and ferromagnetic characteristics in double perovskite semiconductors (DPs) which make the scientific community to further explore these materials for possible spintronic applications.^11^ DPs are compounds with the general formula of ABB′X_6_. Here, A, B, and B′ are the cations, where A generally belongs to rare-earth or alkaline earth metals, B and B′ are transition metals. In the composition, X represents an anion which may be halogen, oxygen, or chalcogenide.^12^ Furthermore, along with their HM and FMS nature, DPs also exhibit optimistic optoelectronic and thermoelectric properties. In the context of thermoelectric (TE) properties, DPs possess high electrical and low thermal conductivities,^13^ high thermal stability, and an environment-friendly nature.^14^

For spintronic devices to work efficiently at room temperature, those HMs are desired which exhibit a large bandgap to avoid spin flipping of carriers from thermal energy and have a high curie temperature (*T*_c_ > room temperature).^15^ In the quest for these properties, Philipp *et al.* reported HM nature in DPs A_2_CrWO_6_ (A = Sr, Ca, and Ba). Among these materials, Sr_2_CrWO_6_ exhibits the highest value of *T*_c_ = 458 K and it showed a tolerance factor equal to 1 which indicates the undistorted cubic structure. The replacement of Ca and Ba in place of Sr causes a reduction in the value of *T*_c_. Furthermore, partial substitution of La^2+^ in place of Sr^2+^ resulted in the reduction in both saturation magnetization and curie temperature.^16^ In another study, Kumar *et al.* reported a half-metallic ferromagnetic HMFM-DP Ba_2_YbTaO_6_, which showed metallic behavior in the spin-down state while exhibiting a wide bandgap of 3.47 eV in the spin-up state.^17^ Besides oxide DPs, to explore the HMFM in halide-based DPs, Mir and Gupta studied Cs_2_NaMCl_6_ (M = V and Ti), and reported the HMFM in both materials with 100% spin polarization. The partial density of states (DOS) plot showed that HMFM properties were contributed by unpaired electrons of split d-orbitals of M-site elements in the crystal field.^18^ In context to TE properties, a material with a high figure of merit (*ZT*) is considered suitable for TE applications. Regarding this, Harbi *et al.* studied Ba_2_MWO_6_ (M = Cd, Zn, and Mg) and reported a high figure of merit at 800 K with the values of 0.64, 0.71, and 0.76 for Cd/Zn/Mg compositions respectively. Also, all three materials were found mechanically stable.^19^ In a separate study about TE properties Haque and Hossain reported a very low lattice thermal conductivity of 0.2 W m^−1^ K^−1^ for the halide DP Cs_2_InAgCl_6_. By increasing temperature from 300 to 700 K power factor decreases but due to low total thermal conductivity, a high *ZT* = 0.74 was obtained at 700 K.^20^ Since the process of doping in DPs affects its TE properties, so to investigate these effects, Hira *et al.*, studied Sr_2−*x*_Bi_*x*_CoRuO_6_ (0 ≤ *x* ≤ 0.8) and reported that with increasing Bi doping, the material changes from n-type to p-type and also a decrease in thermal conductivity was observed. *ZT* value also increased with increasing Bi content and the highest value of *ZT* was reported at *x* = 0.8.^21^

Considering these useful properties of DPs reported in the above-mentioned studies, we investigated the structures of vacancy-ordered DPs Tl_2_WX_6_ (X = Cl, Br) along with their mechanical, electronic, magnetic, and thermoelectric properties by using DFT. The reported results of our study are encouraging and are a worthwhile contribution to the existing knowledge of DPs and also showcased the capability of these materials for application in the field of spintronics and thermoelectrics.

## 2. Computational details

In the present study, we used full potential linearized augmented plane wave (FP-LAPW) calculations for structural optimization and to examine the mechanical, electronic, and magnetic response of Tl_2_WCl/Br_6_. The calculations were performed on the DFT-based Wein2K package. For structural optimization and mechanical properties, exchange correlation potential was evaluated utilizing PBE-sol approximation while for accurate computation of band structure (BS) and density of state (DOS), we utilized mBJ potential. The lattice parameters were calculated by optimization of volume for the lowest ground state energy. To predict the magnetic nature of materials, the volume optimization was implemented separately with initial spin states of antiferromagnetic (AFM) and ferromagnetic (FM) phases. For improved measurements of lattice constants and bulk modulus, the Birch–Murnaghan equation of states was employed.^22^1
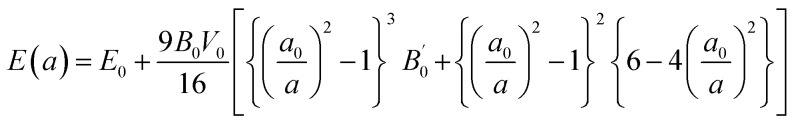


In the above equation, *a*_0_ and *V*_0_ represents the equilibrium lattice constant and volume respectively, *E*_0_ represents energy corresponding to *a*_0_, *B*_0_ and 
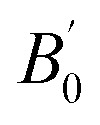
 indicates the values of bulk modulus and its derivative with respect to pressure respectively. To evaluate solution of wave function, both muffin tin (MT) and interstitial regions were examined. In the MT region, we assumed a spherically harmonic solution whereas in interstitial regions, plane wave solution of an electronic system was employed. During the whole calculations, we kept some parameters constant. The constant parameters include the value of angular momentum 
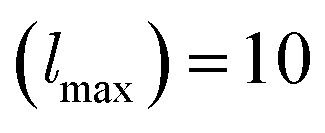
, Gaussian factor *G*_max_ = 20, and the product of MT radius and wave vector (*R* × *K*_max_) = 8. We select a 2000 *k*-point mesh as through iteration, we found that the results start to converge at this value of *k*-points. The energy cutoff was set at 10^−2^ mRy for better convergence. For the computation of thermoelectric properties, semiclassical Boltzmann relations were employed under the fixed relaxation time (*τ* = 10^−14^ s). Boltzmann relations were executed by using BoltzTrap code.

## 3. Results and discussion

### 3.1. Structural properties

**Fig. 1:**
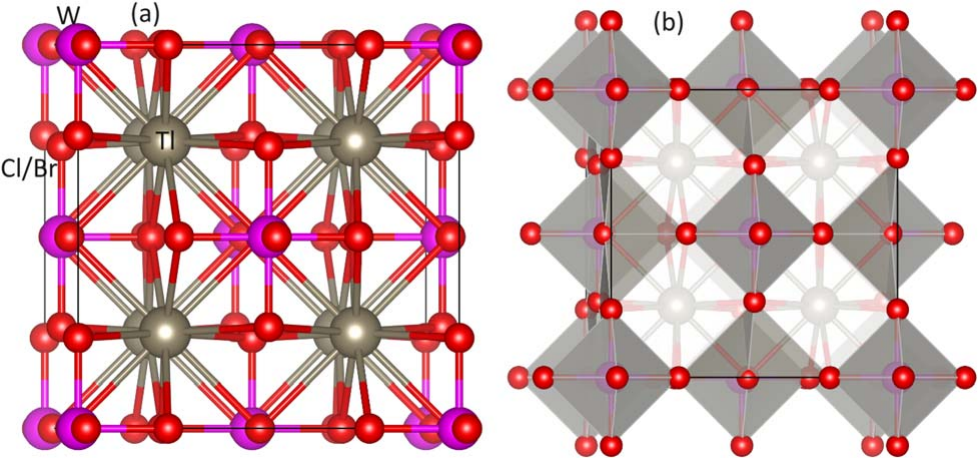
Unit cell of DPs Tl_2_WCl/Br_6_ (a) ball-stick model and (b) polyhedral model. Tl, W, and Cl/Br ions are represented by gray, pink and red spheres respectively.

Unlike normal double perovskites, vacancy-ordered double perovskites have a composition of A_2_BX_6_ in which half of the B site or all the B′ site ions are vacant. Fig. 1(a) shows the ball stick model of Tl_2_WX_6_ (X = Cl, Br) in which Tl, W, and Cl/Br ions are represented by grey, pink, and red color balls respectively. It is clear from the figure that W has a 6-fold coordination with Cl/Br while Tl has a 12-fold coordination with Cl/Br. Another structural model is shown in Fig. 1(b), which represents the polyhedral format of compositions. Due to vacancy ordered composition, alternate B′X_6_ octahedron is not present in the structure leaving behind only the BX_6_ or WCl/Br_6_ octahedron which is highlighted with grey color. Tl cation exists in the space produced by joined neighboring WCl/Br_6_ octahedron. Both of the figures confirm the cubic structure of DPs with the *Fm*3̄*m* space group. The oxidation states of Tl, W, and Cl/Br are +1, +4, and −1 and in both compositions, these ions lie at 8*c*, 4*a*, and 24*e* Wyckoff positions respectively.

**Fig. 2:**
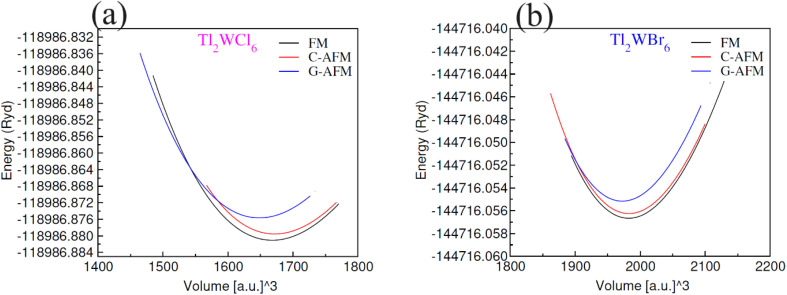
Plots of volume optimization for DPs (a) Tl_2_WCl_6_ and (b) Tl_2_WBr_6_ in FM, C-AFM and G-AFM phases.

The lattice constants *a*_o_ (Å) were calculated by optimization of unit cell volume of compositions for lowest ground-state energy. Fig. 2 shows the volume optimization against ground state energy and the computed values of lattice constant (*a*_o_) are placed in Table 1. The computed value of *a*_o_ for Tl_2_WCl_6_ is 9.96 Å which is well matched with the experimental value.^23^ It is observed that when Cl is replaced by Br the value of *a*_o_ increases and becomes 10.49 Å. This increase in the value of *a*_o_ resulted because of the larger ionic radius of Br^−1^ as compared to Cl^−1^. The magnetic ordering was also investigated through volume optimization by separately taking FM and C/G-AFM spin states as initial approximations. Fig. 2(a) and (b) display that optimization of structure was performed for several magnetic conformations, including ferromagnetic (FM), C-type anti-ferromagnetic (C-AFM), and G-type anti-ferromagnetic (G-AFM). It was found that both DPs phases were stable in ferromagnetic phase. This shows that ferromagnetic ordering is dynamically promising for these states, proposing a particular magnetic configuration that improves their stability. Moreover, energies of formation Δ*H*_f_ were calculated to examine thermodynamic stability. Δ*H*_f_ was given by difference of total energy of a compound and sum of energies of individual elements. The formation energy values are presented in Table 1, which are −1.38 eV and −1.04 eV for the Cl and Br based composition respectively. A negative sign shows that the energy is released during formation which testify to the thermodynamic stability of the compositions. Since the unit cell of Tl_2_WCl_6_ releases more energy during formation than Tl_2_WBr_6_, so the former composition is more stable.

**Table 1:** The optimized lattice constants (*a*_o_), bulk modulus (*B*_o_), the ground-state energy differences between AFM and FM phases, Curie temperature (*T*_c_ (K)) and formation energy (Δ*H*_f_ (eV)) of DPs Tl_2_WCl/Br_6_

DPs	*a*_o_ (Å)	*B*_o_ (GPa)	Δ*E* = *E*_AFM_ − *E*_FM_	*T*_c_	Δ*H*_f_
Tl_2_WCl_6_	9.96	46.25	19.21	558	−1.38
Exp.	9.87 (ref. 23)				
Tl_2_WBr_6_	10.49	37.50	11.06	524	−1.04
Exp.	—				

### 3.2. Mechanical properties

The mechanical stability of the materials was tested by estimating their elastic stiffness constants. Elastic constants help to understand the behavior of materials toward stress. For cubic crystal structure, there are 3 elastic stiffness constants which are *C*_11_, *C*_12_, and *C*_44_. The calculated values of these stiffness constants are listed in Table 2. The mechanical stability of a cubic structure is described by Born stability criteria which states that a cubic structure is stable if *C*_11_–*C*_12_, *C*_11_–2*C*_12,_ and *C*_44_ must be greater than zero.^24^ The calculated constants satisfy these conditions and hence the materials are mechanically stable. Moreover, elastic moduli which include shear modulus (*G*), bulk modulus (*B*_o_) and young modulus (*Y*), were calculated and enlisted in Table 2. It is observed that the values of stiffness constants and elastic moduli are greater in case of Tl_2_WCl_6_ which means that it is more rigid as compared to Tl_2_WBr_6_. The behavior of a decrease in the elastic moduli and elastic stiffness constants, when Cl is replaced by Br, is because of the increase in lattice constants.^25^ Furthermore, in order to check whether the materials are ductile or brittle, Poisson (*υ*) and Pugh (*B*_0_/*G*) ratios were computed. A material is considered ductile if *υ* > 0.26 and *B*_0_/*G* > 1.75.^26,27^ The calculated values of *υ* and *B*_0_/*G* show that both materials are ductile in nature. A ductile or brittle nature can also be confirmed by the term *C*_12_–*C*_44_ called Cauchy pressure. If the Cauchy pressure is positive then material is ductile and it is brittle in case of a negative value.^28^ The positive magnitude of *C*_12_–*C*_44_ confirmed the ductile nature of both compositions. The values of *υ*, *B*_0_/*G*, and *A* are written in Table 2. The anisotropic properties of a material can be evaluated using the formula;
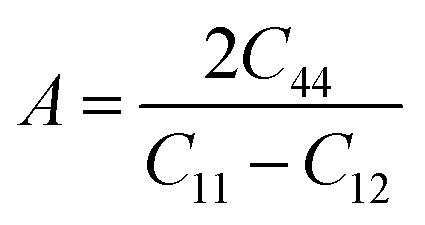


**Table 2:** The computed values of elastic constants (*C*_11_, *C*_12_, *C*_44_), elastic moduli (*B*_0_, *G* and *Y*), *B*_0_/*G*, *υ* and anisotropy (*A*)

DPs	*C*_11_	*C*_12_	*C*_44_	*B*_0_	*G*	*Y*	*B*_0_/*G*	*υ*	*A*
Tl_2_WCl_6_	95.55	20.49	13.38	45.51	20.52	53.53	2.22	0.30	0.36
Tl_2_WBr_6_	82.09	14.90	12.69	37.29	18.97	48.66	1.96	0.28	0.37

In an ideal isotropic system, the value of *A* is one. However, any deviation from this value indicates anisotropic behavior. For the compounds Tl_2_WCl/Br_6_, the calculated *A* values are 0.36 and 0.37, respectively, demonstrating the anisotropic nature of these materials as shown in Table 2.

### 3.3. Electronic properties

**Fig. 3:**
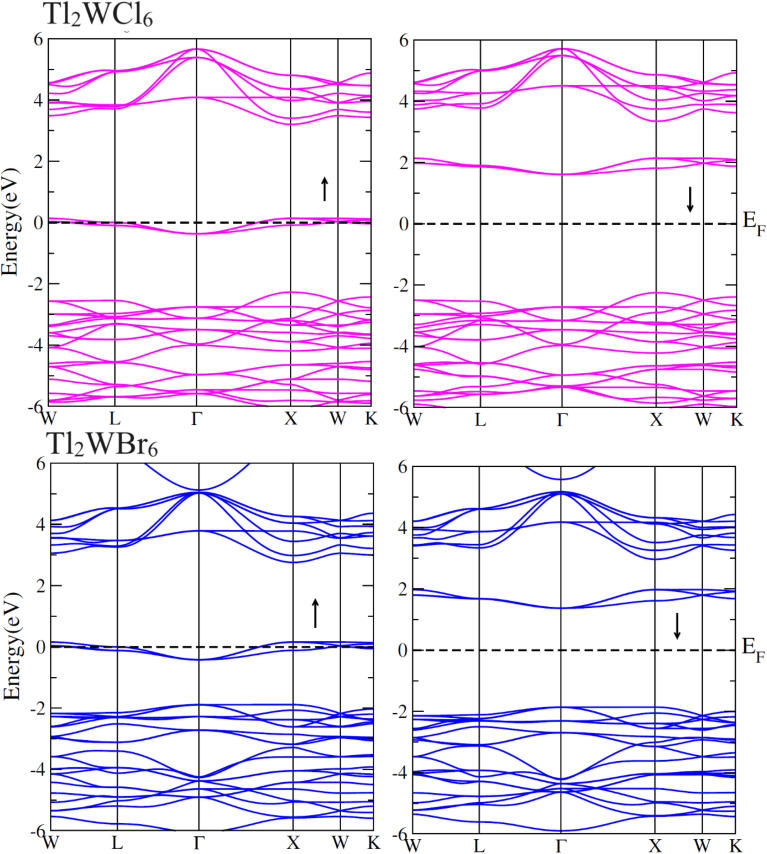
Calculated band structures for DPs Cs_2_W(Cl/Br)_6_ in spin-up (↑) and spin-down (↓) by utilizing mBJ potential.

**Fig. 4:**
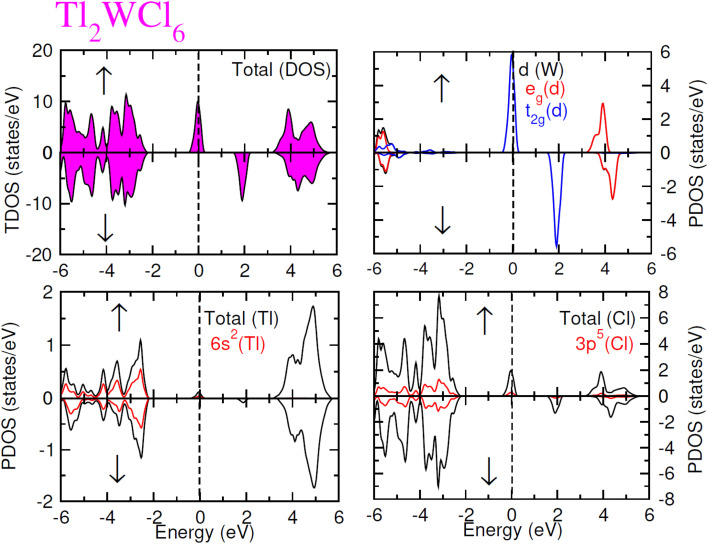
Total DOS and partial DOS for DP Tl_2_WCl_6_ and Tl, W and Cl ions in spin-up (↑) and spin-down (↓) channels by utilizing mBJ potential.

**Fig. 5:**
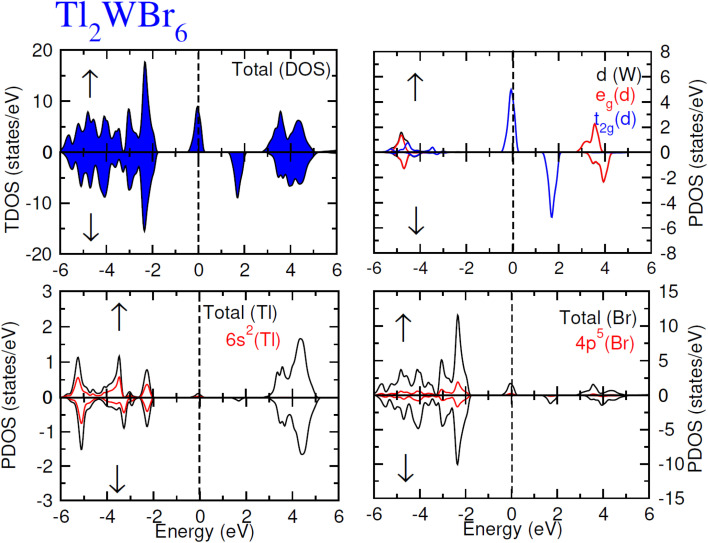
Total DOS and partial DOS for DP Tl_2_WBr_6_ and Tl, W and Br ions in spin-up (↑) and spin-down (↓) channels by utilizing mBJ potential.

The electronic conduction mechanism can be understood by investigating electronic band structure. The band structure was examined through a band structure diagram which is presented in Fig. 3. The band structure diagram of materials was obtained in energy ranges from −6 eV to +6 eV. The spin-polarized band structure confirmed that both materials are halfmetallic in nature. The materials show metallic behavior in up-spin configuration whereas in down-spin configuration, Fermi level lies between the conduction band (CB) and valence band (VB) which shows that in this spin state, the materials are semiconductors. Moreover, the materials exhibit wide bandgaps (*E*_g_) as shown in Fig. 3. In the case of Tl_2_WCl_6_, the *E*_g_ is indirect because the VB maxima and CB minima lie at different K-points. The VB maxima lie at the *X*-symmetry point while the CB minima lie at the *Γ*-symmetry point. Whereas, Tl_2_WBr_6_ exhibits a direct bandgap because the energy maxima of VB and energy minima of CB lie at the same *Γ*-symmetry point. In order to further understand band structure and contribution of different electronic states in the HM nature of materials, total density of states (TDOS) and partial density of states (PDOS) were examined in the same energy range as in the band structure diagram, and their plots are presented in Fig. 4 and 5. TDOS of Tl_2_WCl_6_ and Tl2WBr_6_ as shown in Fig. 4 and 5 in up-spin shows non-zero DOS at the Fermi level which continuously spread across Fermi level demonstrating metallic character while in down-spin, it shows a wide bandgap. HMs are the compounds that generally possess 100% spin polarization which can be computed utilizing given relation:2
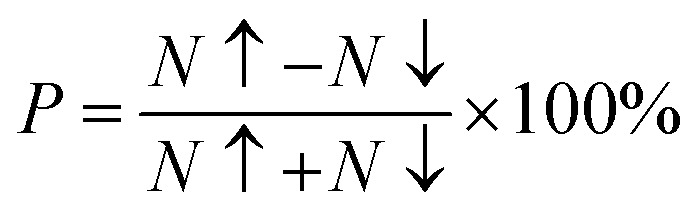
Here, *N*↑ and *N*↓ show the DOS at the Fermi level in spin-up and spin-down respectively.^29^ According to the above relation, studied materials exhibit 100% spin polarization at Fermi level. In Fig. 4. PDOS plots of Tl_2_WCl_6_ show that in this composition, metallic behavior in spin-up is because of major contribution of d-t_2g_ states of W and a minor contribution by the electronic states of Tl and Cl. In spin-down, where the *E*_g_ is present, near the Fermi level, the VB is formed by the major contribution of states of Cl and then Tl and with a very little contribution of d-t_2g_ states of W. In the formation of CB, d-t_2g_ has a vital role while there is a small role of Cl and Tl's states. In the case of Tl_2_WBr_6_, its PDOS illustrated in Fig. 5, shows that d-t_2g_ states of W and electronic states of Br are responsible for metallic behavior in spin-up, with an insignificant contribution of Tl's state also. In spin-down, near Fermi level, VB is formed by a vital role of Br states and a small share of states of Tl while the edges of the CB are majorly shaped by d–t_2g_ states of W with a meager and negligible contribution of electronic states of Br and Tl respectively.

### 3.4. Magnetic properties

To examine the magnetic characteristics of materials, exchange mechanism is crucial to understand. The parameters which are related to the exchange mechanism include crystal field splitting (*Δ*_CF_), exchange splitting (*Δ*_*x*_d), and indirect exchange energy *Δ*_x_(pd). The *Δ*_CF_ arises from the splitting of degenerated d-orbitals of W. When six Cl/Br ligands surround the W cation in an octahedral manner then due to the non-spherical field of ligands, the d-orbitals of W split into three low-energy degenerated t_2g_ orbitals while two high-energy degenerated e_g_ orbitals. *Δ*_CF_ is the difference of energy between these e_g_ and t_2g_ orbitals.^30^ On the other hand, *Δ*_*x*_d can be calculated by the difference of energy among corresponding up-spin and down-spin peaks of d-orbitals.^31^ The values of *Δ*_CF_ and *Δ*_*x*_d are tabulated in Table 3. It can be seen from the results that *Δ*_*x*_d < *Δ*_CF_, and this shows that ferromagnetic nature is dominated in both compositions. The third parameter *Δ*_x_(pd) (which arises from d-states of W and p-states of Cl/Br) can be calculated by taking the difference of energy of valence band maxima in down-spin and up-spin states.^32^

3 *Δ*_x_(pd) = *E*^↓^_v_ − *E*^↑^_v_

Here, *E*^↓^_v_ and *E*^↑^_v_ are the energy of VB maxima in down-spin (↓) and up-spin (↑) respectively. According to the definition of *Δ*_x_(pd), a negative value signifies that the energy of the spin-down state is lower, indicating a preference for downward spin orientation. In ferromagnetism, this preference leads to the alignment of magnetic moments in the same direction, contributing to the overall magnetization of the material.^33^ The values of *Δ*_x_(pd) is listed in Table 3. For further examination of magnetic properties, we calculated s–d and p–d exchange constants *N*_o_*α* and *N*_o_*β* respectively from the difference of energy between down and up-spin states. This energy difference arises from edge splitting at CB minima (Δ*E*_c_ = *E*^↓^_c_ − *E*^↑^_c_) and VB maxima (Δ*E*_v_ = *E*^↓^_v_ − *E*^↑^_v_). The following relations are used for calculations of these constants.4
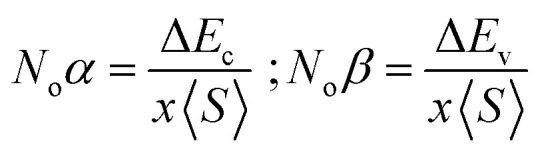
Here, *x* represents the magnetic ion's concentration while *S* represents the mean value of magnetic moment of a magnetic ion. The value of these exchange constants is written in Table 3. The positive magnitude of *N*_o_*α* and negative magnitude of *N*_o_*β* are obtained which is a typical response in HMFM semiconductors.^33^

**Table 3:** The estimated magnitudes of *Δ*_CF_, *Δ*_x_(d), *Δ*_x_(pd), *N*_o_*α* and *N*_o_*β* for DPs Tl_2_WCl/Br_6_

DPs	*Δ*_CF_	*Δ*_x_(d)	*Δ*_x_(pd)	*N*_o_ *α*	*N*_o_ *β*
Tl_2_WCl_6_	5.8	4.2	−2.2	0.42	−2.93
Tl_2_WBr_6_	5.4	3.9	−1.9	0.31	−2.56

The total magnetic moment along with local magnetic moment of Tl, W and Cl/Br were calculated and enlisted in Table 4. It is observed that both compositions have the same value for total magnetic moment with a major contribution from W ions while Tl and Cl/Br contribute insignificantly to net magnetic moment. Since the magnetic moment in an energy range arises from spin polarization which depends on the difference of integral of spin DOS in that range of energy, so, the exchange splitting induces magnetic moment. The large exchange splitting in d-orbitals of W is accountable for its larger magnetic moment as this makes one spin state favorable below the Fermi level. Moreover, the interstitial magnetic moment was also calculated and it has a decent share in the net magnetic moment. The interstitial magnetic moment values are given in Table 4. Since for spintronic devices to work at room temperature, their Curie temperature (*T*_c_) must be higher than room temperature. The Curie temperature (*T*_c_) has been computed by utilizing smearing in mean-field approximations, stated as 
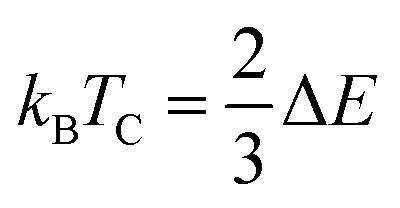
.^31^ The magnetic inter-connection between two W atoms is described as difference of energy between FM and AFM states Δ*E* = *E*_FM_ − *E*_AFM_. The calculated values of *T*_c_ for studied materials were obtained very large as compared to room temperature. The *T*_c_ values are given in Table 1 as 558 and 524 K for Tl_2_WCl_6_ and Tl_2_WBr_6_ respectively. The HMFM nature of these materials with a wide *E*_g_ in minority spin channels and larger *T*_c_ make them potential candidates for practical spintronic applications.

**Table 4:** Magnetic moments value for DPs Tl_2_WCl/Br_6_, total and their local *M*_Tl_, *M*_W_, and *M*_Cl/Br_, interstitial (*M*_Int._)

DPs	*M*_Total_	*M*_Int._	*M*_Tl_	*M*_W_	*M*_Cl/Br_
Tl_2_WCl_6_	2.000	0.451	0.001	1.488	0.009
Tl_2_WBr_6_	2.000	0.487	0.002	1.481	0.004

### 3.5. Thermoelectric properties

All the energy generation processes are not 100% efficient and a great amount of energy in the form of heat is wasted in the environment. In this situation, thermoelectric (TE) devices provide an eco-friendly way to convert this wasted heat into electrical energy. In recent decades, materials that possess TE characteristics have been studied extensively. In this article, we reported TE properties of Tl_2_WCl_6_ and Tl_2_WBr_6_ calculated by the BoltzTrap code. The reported TE properties include Seebeck coefficient *S*, thermal and electrical conductivities per relaxation time (*κ*_e_/*τ*) and (*σ*/*τ*) respectively, magnetic susceptibility (*χ*), power factor (P.F.), and figure of merit (*ZT*) in the range of temperature from 200–800 K. Due to its half-metallic nature, these parameters give different results in different spin channels, so the net effect of these parameters can be calculated using following expressions.^34^5
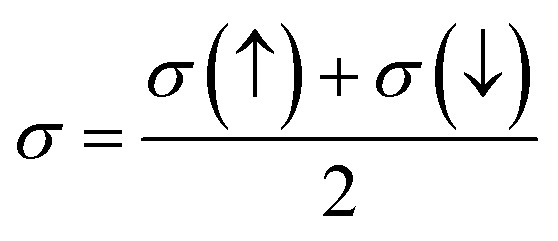
6
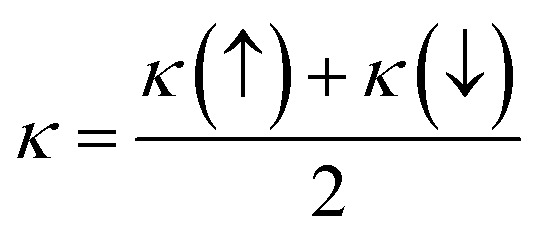
7
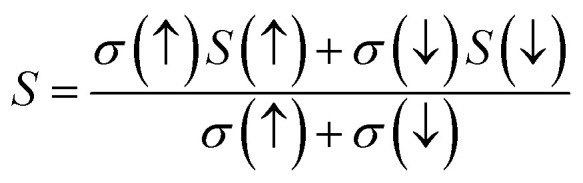


**Fig. 6:**
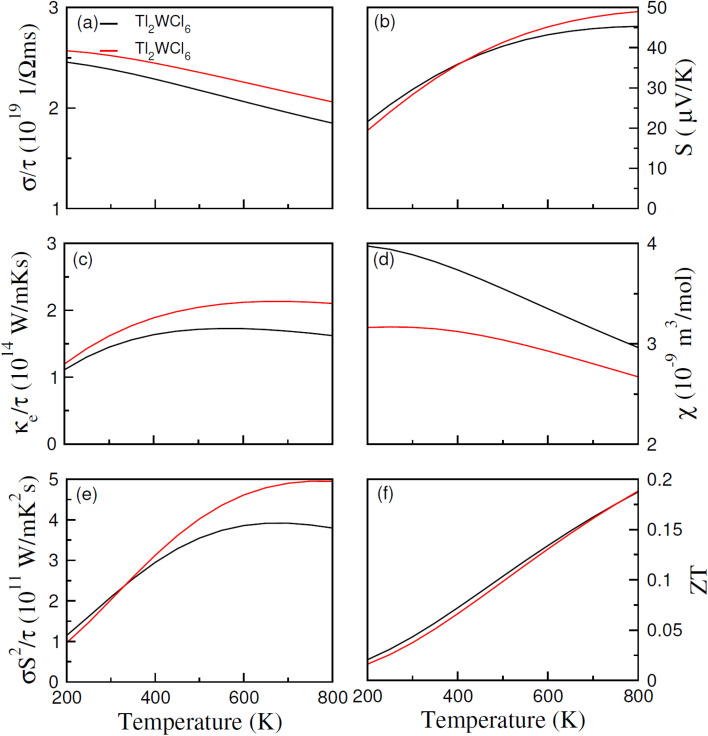
The calculated values of (a) *σ*/*τ*, (b) *S*, (c) *k*_e_/*τ*, (d) *χ*, (e) power factor and (f) *ZT* against temperature for DPs Tl_2_W(Cl/Br)_6_ using BoltzTrap code.

In Fig. 6(a), (*σ*/*τ*) for both compositions are observed to decrease with an increase in temperature. At 200 K their values are 2.45 × 10^19^ and 2.6 × 10^19^ (Ω ms)^−1^ for Cl and Br-based composition which decreases with temperature and becomes 1.8 × 10^19^ and 2.13 × 10^19^ (Ω ms)^−1^ at 800 K respectively. The decrease in the (*σ*/*τ*) arises because of the HM nature of materials with wide *E*_g_ as (*σ*/*τ*) in spin-up decreases due to metallic nature in that spin channel while in spin-down, owing to wide *E*_g_, the carrier density cannot increase significantly causing net (*σ*/*τ*) to decrease. The total thermal conductivity (*κ*) is the sum of thermal conductivity contributed by charge carriers (*κ*_e_) and lattice vibrations or phonons (*κ*_L_). In this work, we only computed *κ*_e_, and their values were displayed in Fig. 6(c). At 200 K, Tl_2_WCl_6_ and Tl_2_WBr_6_ have the (*κ*_e_/*τ*) of 1.1 × 10^14^ and 1.2 × 10^14^ W m K^−1^ s^−1^ respectively which first increases non-linearly with temperature and then start to decrease and gives the final values of 1.65 × 10^14^ and 2.15 × 10^14^ W m K^−1^ s^−1^ at 800 K respectively for Tl_2_WCl_6_ and Tl_2_WBr_6_. The ratio (*κ*_e_/*σ*) is found to be in the order of 10^−5^ making it a suitable candidate for TE applications. Another important parameter is Seebeck coefficient (*S*) which measures magnitude of potential difference induced at the ends of TE material when it is subjected to temperature gradient. The magnitude of *S* can be estimated by using the Pisarenko relation.^35^8
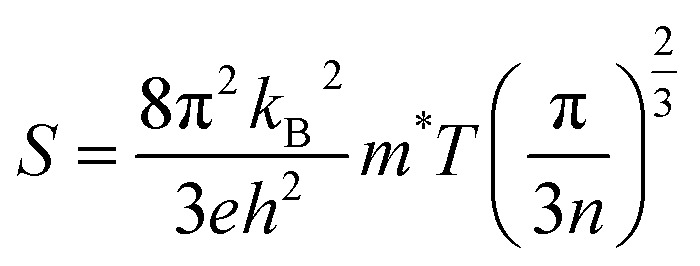
Here, the symbols *e*, *h*, *k*_B_, *T*, m*, and *n* represent the carrier's charge, Plank's constant, Boltzmann constant, the absolute value of temperature, effective mass of charge carrier, and carrier concentration respectively.

It is observed from Fig. 6(b), that *S* increases for both materials when temperature is increased. The estimated values of *S* at 200 K are 19.5 and 21.5 μV K^−1^, which at 800 K becomes 45 and 49 μV K^−1^ for Tl_2_WCl_6_ and Tl_2_WBr_6_. This increasing behavior is because of the direct relation between *S* and *T*. Moreover, positive magnitude of *S* shows majority of positive charge carriers. To analyze effect of temperature on the magnetization of the materials, their magnetic susceptibility (*χ*) against temperature range of 200–800 K was calculated and presented in Fig. 6(d). Magnetic susceptibility is the measure of the magnetization of materials in the existence of applied magnetic field. It is noted from figure that *χ* decreases by increasing temperature which is a typical response in the case of ferromagnetic materials and follows the Curie Weiss law. When temperature increases, the thermal energy disrupts the alignment of domains, which causes a decrease in magnetization which in turn decreases the *χ*. In order to probe how efficiently, a TE material works and convert thermal energy into electrical energy, a parameter named power factor (P.F.) was calculated. The power factor was calculated by the relation P.F. = *σS*^2^/*τ* and its values are demonstrated in Fig. 6(e). At 200 K, P.F. value is 1.2 × 10^11^ and 1.0 × 10^11^ W mK^−2^ s^−1^ for Tl_2_WCl_6_ and Tl_2_WBr_6_ respectively. An increasing trend is observed in the P.F. for both materials and at 800 K it becomes 3.7 × 10^11^ W mK^−2^ s^−1^ for Cl-based material and 5 × 10^11^ W mK^−2^ s^−1^ for Br-based material. Lastly, the overall performance of any TE material can be assessed by an ultimate dimensionless quantity called figure of merit (*ZT*). The *ZT* value can be measured using the relation *ZT* = (*S*^2^*σ*/*κ*)*T* and its computed values are illustrated in Fig. 6(f). In the case of Tl_2_WCl_6_, at 200 K the computed *ZT* is 0.02 which increases almost linearly and becomes 0.185 at 800 K while in the case of Tl_2_WBr_6_, the value of *ZT* is 0.016 and 0.185 at 200 K and 800 K respectively.

**Fig. 7:**
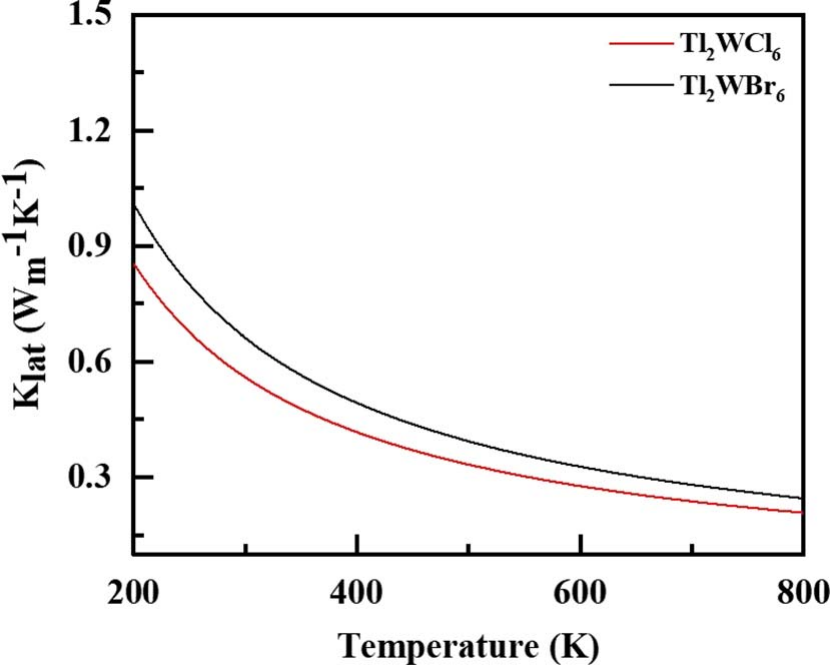
The calculated lattice conductivity (*k*_lat_/*τ*) for DPs Tl_2_W(Cl/Br)_6_ against temperature 200–800 K.

To accurately determine the actual values of *ZT*, it is essential to explain lattice thermal conductivity (*κ*_L_). Therefore, *κ*_L_ is computed separately and shown in Fig. 7 as a function of temperature (*T*).^36^ The low *κ*_L_ values for both compositions indicate that its contribution is minimal, which is advantageous for enhancing the performance of these materials. Specifically, the *κ*_L_ value for Tl_2_WCl_6_ is reported as 0.85 and for Tl_2_WBr_6_ is 1.03 W m K^−1^ at 200 K. These values decrease to 0.21 and 0.27 W m K^−1^ at 800 K, respectively, highlighting the significant contribution of *κ*_L_ to the overall value of thermal conductivity of these materials.^37^

### 3.6. Phonon dispersion plot

**Fig. 8:**
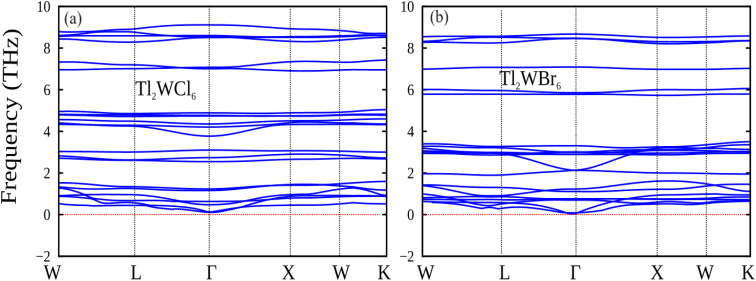
The calculated phonon dispersion plot for DPs (a) Tl_2_WCl_6_ and (b) Tl_2_WBr_6_.

Fig. 8 presents the phonon band spectra for the double perovskites Tl_2_WX_6_ (X = Cl and Br), which is computed using the PBE-GGA approximation.^38^ Each double perovskite structure, comprising 9 atoms, yields a total of 27 phonon modes. These modes are categorized into 3 acoustic and 24 optical phonons. The positive frequency values confirm the stability of the studied double perovskite compositions.^39^

## 4. Conclusion

In this study, we comprehensively explored DPs Tl_2_WX_6_ (X = Cl and Br) for their electronic, magnetic, and thermoelectric properties by utilizing DFT. The materials were found to be mechanically stable and ductile as suggested by their computed elastic constants. The negative formation energies (Δ*H*_f_) testify that both materials were thermodynamically stable. The values of Δ*H*_f_ for Tl_2_WCl_6_ and Tl_2_WBr_6_ were −1.38 and −1.04 eV respectively, which indicates that the former one is more stable with more energy released at the time of formation. Volume optimization showed that materials with ferromagnetic spin states have the lowest energies which indicated that they are more stable in the ferromagnetic phase than the antiferromagnetic phase. The band structure diagram and DOS depicted that the materials are halfmetallic and at Fermi level they exhibited 100% spin polarization. Both compositions exhibited metallic character in up-spin configuration and a wide bandgap in down-spin states. Moreover, (+ve) values of *N*_o_*α* and (−ve) values of *N*_o_*β* give further evidence of halfmetallic ferromagnetic nature. Moreover, a high *T*_c_ for Tl_2_WCl_6_ and Tl_2_WBr_6_ was obtained with values of 558 and 524 K which are well above the room temperature. In regards to thermoelectric (TE) properties, electrical conductivity (*σ*/*τ*), and magnetic susceptibility (*χ*) were found to decrease with temperature while other TE parameters which include, Seebeck coefficient (*S*), electronic part of thermal conductivity (*k*_e_/*τ*), power factor (P.F.), and figure of merit (*ZT*) increases with temperature. The calculated *ZT* for both materials were 0.185 at 800 K. The halfmetallic ferromagnetism with a high curie temperature and wide bandgap along with TE characteristics make these materials potential candidates for spintronic and TE applications.

## Conflicts of interest

There are no conflicts to declare.

## Supplementary Material
